# Prevalence of *Trypanosoma evansi* in livestock in Palestine

**DOI:** 10.1186/s13071-020-3894-9

**Published:** 2020-01-13

**Authors:** Suheir Ereqat, Abdelmajeed Nasereddin, Amer Al-Jawabreh, Hanan Al-Jawabreh, Nahed Al-Laham, Ziad Abdeen

**Affiliations:** 1 Al-Quds University, East Jerusalem, Palestine; 2 Al-Quds Public Health Society, Jerusalem, Palestine; 3 Arab American University, Jenin, Palestine; 4 Leishmaniases Research Unit, Jericho, Palestine; 5 Department of Laboratory Medicine, Faculty of Applied Medical Sciences, Al-Azhar University-Gaza, Gaza Strip, Palestine

**Keywords:** *Trypanosoma evansi*, Camel, Surra, Palestine, PCR, Trypomastigote

## Abstract

**Background:**

*Trypanosoma evansi* is the causative agent of surra, a disease that occurs in many animal species. The disease is responsible for substantial losses in global production and can be fatal if not diagnosed early. This study aims to determine the prevalence of *T. evansi* in livestock, equids and dromedary camels in Palestine.

**Methods:**

Blood samples were collected during 2015–2017 from domesticated animals (*n* = 259 animals; 77% females and 23% males) including camels (*n* = 87), horses (*n* = 46), donkeys (*n* = 28), mules (*n* = 2), sheep (*n* = 49) and goats (*n* = 48) from eight districts: Ariha (Jericho), Nablus, Bethlehem, Deir Al Balah, Jenin, Rafah, Tubas, and Khan Yunis. Parasite prevalence was determined using PCR and blood smear microscopy. PCR-positive samples were further phylogenetically analyzed using DNA sequences of the *18S* ribosomal RNA gene.

**Results:**

The overall infection prevalence was 18% (46/259). The positivity rates according to PCR and microscopy examination were 17% (45/259) and 2.7% (7/259), respectively. The infection rates were as follows: camels, 26/61 (30%); horses, 8/46 (17%); donkeys, 3/28 (11%); mules, 1/2 (50%); sheep, 2/42 (4%); and goats, 6/42 (13%). Phylogenetic analyses of the *18S* rRNA gene showed that 24 positive *T. evansi* samples from Palestine formed a monophyletic cluster with seven *T. evansi* sequences from Africa, Asia and South America, and three *T. brucei* sequences from Africa retrieved from GenBank. The spatial analysis showed three statistically significant foci of *T. evansi* infection in Jenin, Tubas (*P* = 0.02) and Ariha (Jericho) (*P* = 0.04). No statistically significant foci were detected in the Gaza Strip.

**Conclusions:**

To the best of our knowledge, this is the first confirmation of high levels of infection with *T. evansi* as a causative agent of surra in Palestine. Our study emphasizes the need for a stringent surveillance system and risk assessment studies as prerequisites for control measures. Further investigations focusing on vectors and evaluation of risk factors are needed.

## Background

Surra, or trypanosomiasis in vertebrate animals, is caused by *Trypanosoma evansi*, a salivarian protozoan of the family Trypanosomatidae. It is hypothesized that *T. evansi* initially developed in camels and has since spread to many domestic and wild mammals [1–10]. Recent studies have reported atypical human *T. evansi* infection related to a lack of apolipoprotein L-I [11, 12]. Hematophagous horseflies of the genus *Tabanus* and stable flies of the genus *Stomoxys* [6] seem to play an important role in the mechanical transmission of *T. evansi*; transmission can also occur through the contamination of a wound with infected animal blood [13]. The wide range of parasite hosts contributed to its geographical spread in 48 countries throughout the world [6, 14–17]. The disease can cause significant economic damage mainly due to reduced milk yields, decreased animal market values and annual mortality rates affecting thousands of animals [4, 18, 19]. The clinical manifestations of trypanosomiasis in animals are influenced by both the host and *Trypanosoma* species. In general, the disease is fatal unless treated and can cause a wide range of symptoms in different animals [6].

Several methods for the detection of *T. evansi* infection have been developed. Serology, such as enzyme-linked immunosorbent assay (ELISA), can be used to monitor animals on a large scale but may present cross-reactions between different species of trypanosomes. Furthermore, it cannot differentiate between sick and cured animals. Blood smear examination, which is a simple, inexpensive and quick method, is known to be of limited sensitivity [20], and it can be difficult to detect parasites in the early stages of infection due to low levels of parasitemia. The *T. evansi* diagnostic stage, the trypomastigote, is among the few blood parasites that can be viewed by direct wet mount due to its vigorous motility. Polymerase chain reaction (PCR)-based techniques have been described as the most accurate tools for the diagnosis of subclinical and latent infections [21]. Several molecular-based methods targeting different genes with varying degrees of sensitivity and specificity have been described for the detection and identification of trypanosomes. Previous studies have indicated that the RoTat 1.2 variable surface glycoprotein (VSG) gene is present in all *T. evansi* strains, except for some Kenyan strains, and therefore can be used as a specific marker for *T. evansi* [22, 23]. Surra disease was previously reported in countries neighboring Palestine. In Jordan, the seroprevalence in camels and horses was reported to be 30.5% and 33.3%, respectively [24]. In areas of Israel near the Dead Sea and Wadi Araba region, the infection rate in horses based on molecular methods was reported to be 18.7%. In 2010, an outbreak of trypanosomiasis caused by *T. evansi* was identified in a farm in the same geographical area, demonstrating differences in infection susceptibility between different species of animals, such as camels (80%), horses (43%) and donkeys (46%) [22, 25, 26]. In Ismailia, Egypt, the prevalence in camels ranged from 10% to 46% [27]. In Saudi Arabia, variable infection rates were reported in horses (3.3%) donkeys (2.8%) and camels (5–40%) [28–30]. In Iran, the infection rate ranged from 0 to 19.5% in camels [31–33].

This study aimed to determine the frequency of *T. evansi* in Palestine by employing molecular and light microscopy methods. For further epidemiological investigations, phylogenetic and spatial analyses were also used.

## Methods

### Study design and sample collection

A cross-sectional study was conducted between 2015 and 2017. A standard questionnaire that included several variables, such as sex, age, location, and clinical signs, was completed for each animal. Based on availability and the herd owners’ approval, convenience sampling of camel herds in the West Bank and Gaza Strip was conducted; in addition, horses, mules, donkeys, goats and sheep that were close to the camel herds were also sampled. Peripheral blood samples (*n* = 259) were obtained from the jugular vein from each animal using 5 ml disposable syringes and collected in tubes containing ethylenediaminetetraacetic acid (EDTA) for further parasitological diagnosis and PCR. The samples were immediately sent to the laboratory for processing.

### Direct wet mount and Giemsa-stained thin smears

All samples were screened by direct wet mount examination of an EDTA blood drop, and only positive wet mount samples examined as Giemsa-stained thin blood smears. Wet-mount smears were prepared according to Garcia [34, 35] with slight modifications. In brief, the EDTA blood tube was gently mixed. Five microliters of EDTA whole blood was placed in the middle of a clean slide. Ten microliters of warm normal saline was added and mixed; the mixture was then covered with a coverslip and examined under a microscope at a magnification of 400×. Dilution was necessary to establish evenly distributed unstacked erythrocytes, make the actively motile trypomastigotes readily observable and prevent otherwise stacked RBCs from masking the parasites in the case of light infections. A coverslip of 22 × 22 mm was completely scanned before declaring a negative result. Thin blood films were prepared from positive wet mounts, stained by Giemsa stain and examined for confirmation. In brief, 10 µl of EDTA whole blood was placed on one side of a clean slide and spread as a thin film. The film was allowed to dry for 1 h and then fixed with absolute methanol for 3 min. Subsequently, the methanol was tapped off and the thin smear was immersed in a Coplin Jar filled with 1:20 diluted Giemsa stain. The blood film was stained for 20 min. The blood film was observed under magnifications of 400× and 1000× (oil immersion microscopy) to identify trypomastigotes.

### DNA extraction

DNA was isolated from 200 µl EDTA whole blood using QIAamp® DNA mini and blood mini kits (Qiagen, Hilden, Germany) according to the manufacturer’s instructions. The DNA was stored at − 20 °C until use.

### DNA amplification

Conventional PCR was used for the detection of *T. evansi* DNA based on species-specific primers targeting 257 bp of the trypanosome-specific repetitive nucleotide sequence of the RoTat 1.2 VSG gene, as previously described [22, 25, 26, 36], with some modifications. PCR amplification was performed using a Biometra T Advanced instrument (Analytik Jena AG, Jena, Germany). The primer pair TR3 (5’-GCG CGG ATT CTT TGC AGA CGA-3’) and TR4 (5’TGC AGA CAC TGG AAT GTT ACT-3’) at a concentration of 10 µM per primer in a total reaction volume of 25 µl using PCR-Ready Supreme mix (Syntezza Bioscience, West Jerusalem, Israel) was used. PCR mix with distilled water was used as a negative control. The PCR cocktail was heated at 95 °C for 5 min; followed by 35 cycles of 30 s at 95 °C, 40 s at 55 °C, and 45 s at 72 °C; and a final extension step at 72 °C for 7 min. The amplicons were visualized by electrophoresis using a 2% agarose gel stained with ethidium bromide.

### DNA sequencing of the *18S* rRNA gene and phylogenetic analysis

All positive samples according to VSG3 PCR were further evaluated for genotype by analyzing a 235-bp fragment of the *18S* small subunit ribosomal RNA (rRNA) gene using two primers (18S3F: 5’-GAC CRT TGT AGT CCA CAC TG-3’ and 18S4R: 5’-CCC CCT GAG ACT GTA ACC TC-3’), as previously described [37–39]. PCR was performed in a 25 µl total reaction volume including 0.6 µM of primers and 5 µl of DNA template. The following PCR conditions were used: 95 °C for 5 min; followed by 35 cycles of 30 s at 95 °C, 35 s at 60 °C and 45 s at 72 °C; and a final extension step at 72 °C for 7 min. The positive PCR samples were sent for commercial bidirectional Sanger DNA sequencing, and the sequences were assembled by using online multiple sequence software Multialin (http://multalin.toulouse.inra.fr/multalin/) by Corpet [40]. Phylogenetic trees of the *18S* rRNA sequences were constructed by the unweighted pair group method with arithmetic mean (UPGMA), neighbor-joining and maximum likelihood algorithms. The phylogenetic tree was conducted with MEGA X software [41] using the UPGMA program. The reliability of internal branches was assessed by bootstrapping with 1000 pseudoreplicates. Nodes with bootstrap support of < 70% were collapsed. The following GenBank sequences were included in the analysis: *T. equiperdum* isolate MP77 (KY609968.1); *T. evansi* isolate DH4 (KY114580.1); *T. evansi* isolate CB2 (KY114579.1); *T. evansi* isolate Egy.4 (AB551922.1); and *T. evansi* isolate T4 (KT844946.1).

### Statistical and spatial analyses

The Epi Info™ statistical package (CDC free software) was used for frequency analyses, Chi-square tests, Fisher’s exact tests, *post-hoc* pairwise Fisher’s exact tests, and spot- and cluster mapping of *Trypanosoma* smear results or positive cases according to PCR. SaTScan^TM^ v8.0 freeware was used to detect statistical evidence for pure spatial clustering of *T. evansi* cases. The analysis was performed at a district-wide level. The SaTScan analysis is based on scanning a window across space [42]. For each location and size of the window, the observed and expected number of cases is compared to those outside of the window. The window with the greatest ratio of observed-to-expected cases is indicated on the map. The window identified as least likely due to chance is subsequently evaluated by a maximum likelihood ratio test with a test decision based on a Monte-Carlo simulated *P*-value (999 simulations). The maximum proportion of the population that a cluster could contain was set at 50% of the cases, without geographical overlap. The data were analyzed based on discrete Poisson model scanning for areas with significantly high rates of infection, with *P*-values ≤ 0.05.

## Results

**Fig. 1:**
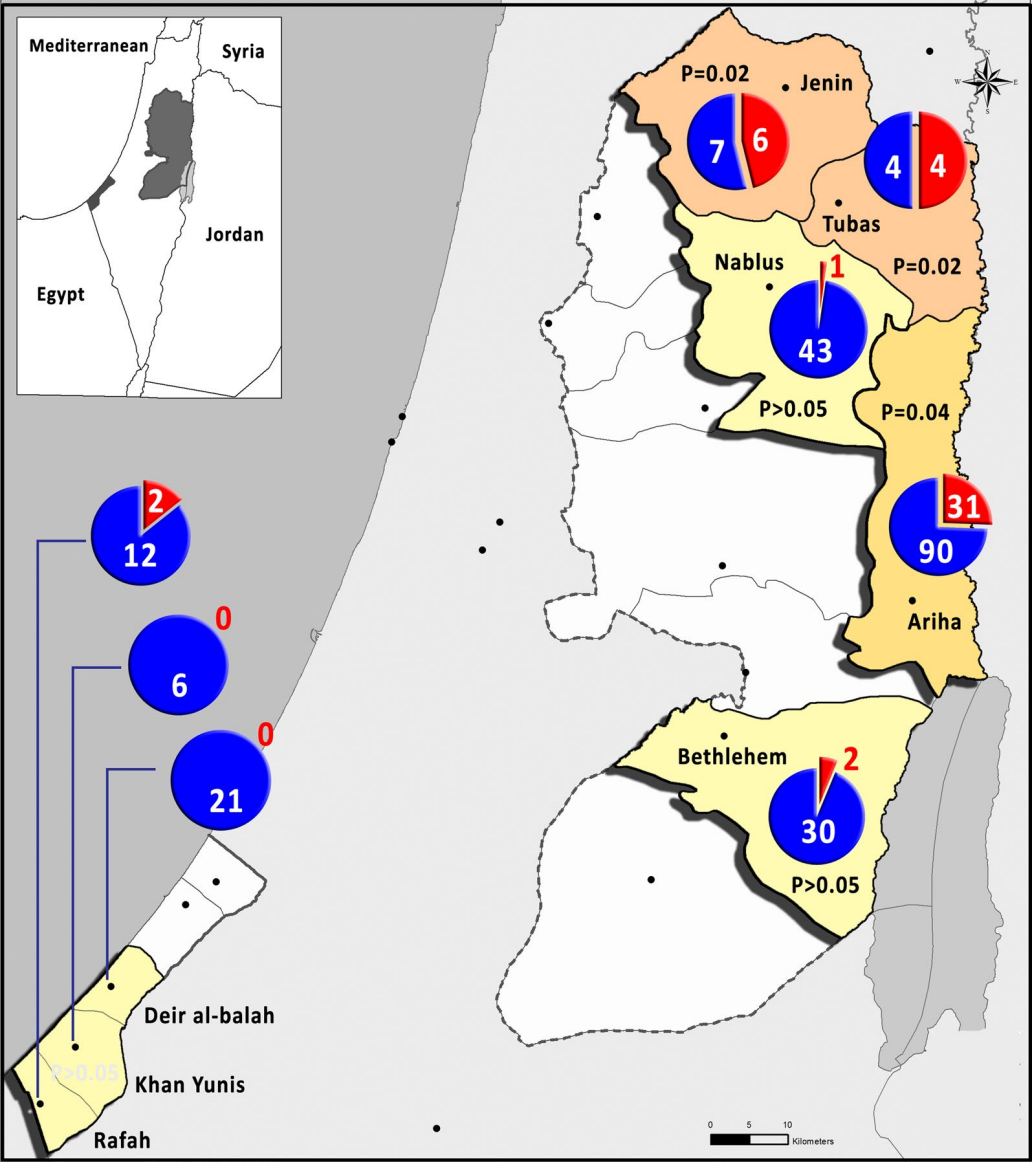
Cluster map of *T. evansi* cases in Palestine, the West Bank and the Gaza Strip. The red color in the pie chart represents the positive cases, while the blue color represents the negative cases. The districts of Jenin, Tubas and Ariha contained significant *Trypanosoma* clusters (*P* < 0.05)

A total of 259 animals were examined for infection with *T. evansi* using parasitological and molecular methods. The overall infection prevalence was 18% (46/259), with different rates in different animal species: camels (*n* = 87), sheep (*n* = 49), goats (*n* = 48), horses (*n* = 46), donkeys (*n* = 28) and mules (*n* = 2) (Tables 1, 2). Among the total, female animals were dominant, at 78% (201/259). Samples positive by microscopy and PCR analysis were 2.7% (7/259) and 17% (45/259), respectively. Six of the seven positive cases by wet mount examination were also PCR positive (86%). A goat showing few parasites per high power field (400× magnification) was the only positive wet mount with a PCR-negative sample. PCR targeting the RoTat 1.2 VSG gene revealed 45 positive cases, of which 35 were also positive by *18S* rRNA gene amplification. However, only 24 were successfully sequenced. Trypanosomiasis was detected across all six species of livestock included in this study, with a significant difference among them (*χ*^2^ = 18.7, *df* = 5, *P* = 0.002) (Table 1). *Post-hoc* pairwise Fisher’s exact tests showed that camels (*P* < 0.00001) and sheep (*P* = 0.0034) had significantly higher rates of infection than the other species. A pure spatial analysis at the district level revealed three significant clusters: Jenin, Tubas and Ariha (Jericho) (Fig. 1). All three significant foci were in the West Bank, while the Gaza Strip was free from any significant clusters. The infection rate in the West Bank was 20% (44/218) compared to 5% (2/41) in the Gaza Strip.

**Table 1:** PCR and microscopy results of the 259 tested animals

		PCR		
		Positive	Negative	Total
Microscopy	Positive	6	1	7
	Negative	39	213	252
	Total	45	214	259

**Table 2:** Trypanosomiasis by animal species as detected by either PCR or wet mount smears

Animal species	No. positive (%)	No. negative	Total	*P*-value^a^
Camel	26 (30)	60	86	< 0.00001
Horse	8 (17)	38	46	1
Donkey	3 (11)	25	28	0.43
Mule	1 (50)	1	2	0.32
Sheep	2 (4)	47	49	0.0034
Goat	6 (13)	42	48	0.4
Total	46 (18)	213	259	

^a^Fisher’s exact test

*Note*: Chi-square followed by *post-hoc* pairwise Fisher’s exact tests, with a level of significance at < 0.05

In this cross-sectional study, sex and age group were not significant risk factors for infection in livestock, as shown in Table 3.

**Table 3:** Prevalence of *Trypanosoma evansi* infection in animals by sex and age group based on the PCR and wet mount results

Variable	State	No. infected	No. uninfected	OR (95% CI)	*P*-value^a^
Sex	Male	35	166	0.9 (0.4–1.9)	1
	Female	11	47		
Age group (years)	< 2	0	5	0.53 (0.03–10.3)	0.8
	> 2	8	49		

^a^ Fisher’s exact test

*Abbreviations*: OR, odds ratio; CI, confidence interval

**Fig. 2:**
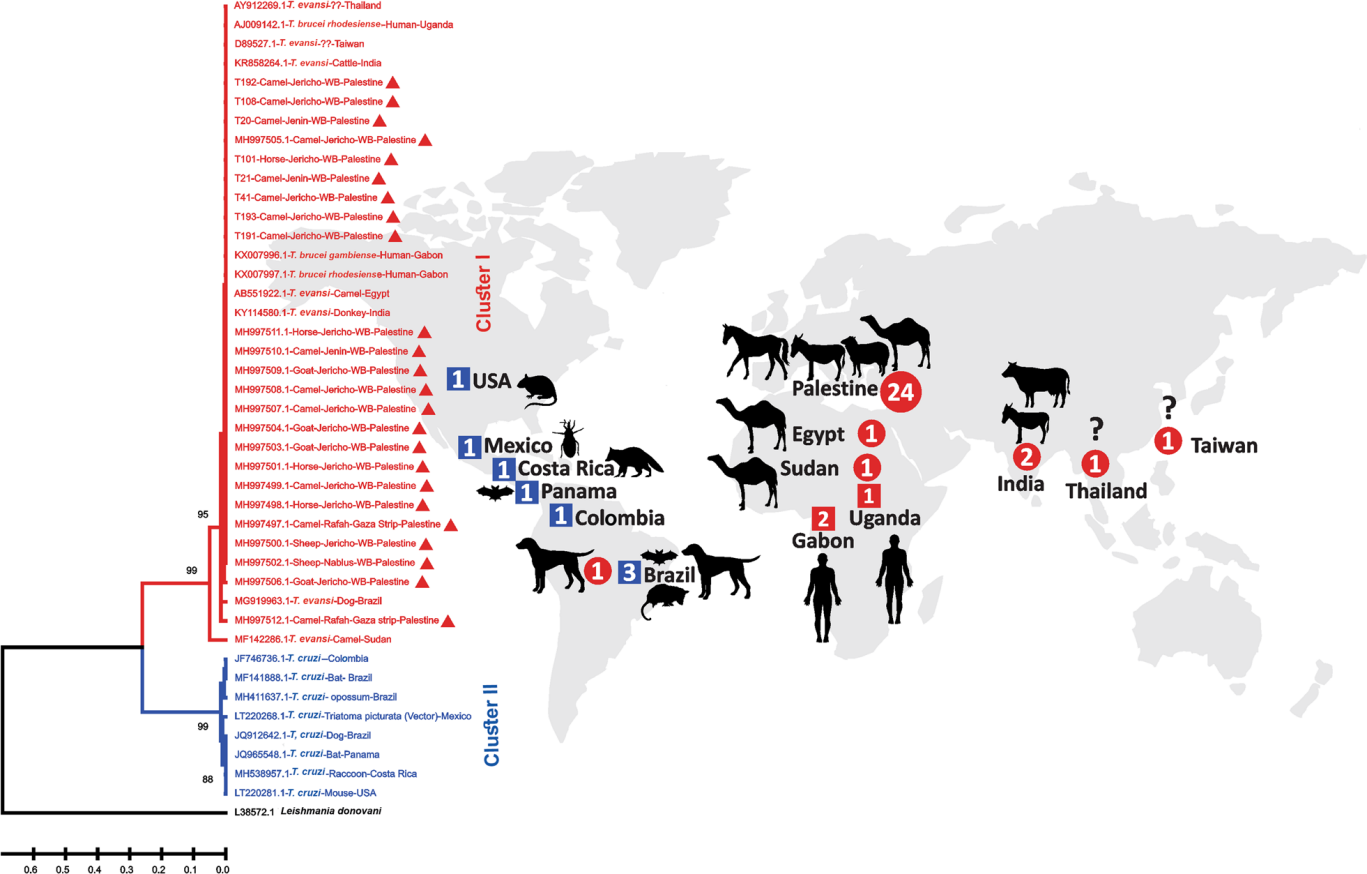
Phylogenetic tree based on partial DNA sequences of the *18S* rRNA gene (237 bp) showing the relationship between the Palestinian *T. evansi* samples (*n* = 24) and the *T. evansi* sequences from Africa (*n* = 2), Asia (*n* = 4) and South America (*n* = 1) and *T. brucei* sequences (*n* = 3) from Africa on GenBank. The Palestinian strains are designated by red triangles. The red circles on the world map represent *T. evansi*, the red squares represent *T. brucei*, and the blue squares represent *T. cruzi*. The tree was constructed using the statistical UPGMA algorithm [43]. The numbers next to the branches represent the percentage of bootstrap values based on 1000 replicates [44]. The branch length scale is shown below the tree, indicating the evolutionary distance that was computed based on the maximum composite likelihood method; the unit is the number of base substitutions per site [41]. The tree was constructed using MEGA X [41]. *Leishmania donovani* from Sudan (MHOM/SD/00/Khartoum; GenBank: L38572.1) was used as the outgroup

The phylogenetic analysis using the *18S* rRNA gene showed two clusters with significant bootstrap values (Fig. 2). Cluster I included *T. evansi* from Asia, Africa and South America isolated from dogs, donkeys, camels, sheep, goats, horses and cattle. All of the *T. evansi* sequences that grouped with *T. b. gambiense* and *T. b. rhodesiense* from Africa were isolated from human hosts. Cluster II consisted exclusively of *T. cruzi* from Latin and North America. All *T. evansi* (*n* = 24) from Palestine (the West Bank and Gaza Strip) clustered in Cluster I (Fig. 2). The Palestinian *18S* rDNA sequences were deposited in the GenBank database under the accession numbers MH997497-MH997512.

The pure spatial analysis showed statistically significant foci of *T. evansi* infection in three main districts: Jenin, Tubas (*P* = 0.02) and Ariha (Jericho) (*P* = 0.04) (Fig. 1). No statistically significant foci were detected in the Gaza Strip. The infection rate was significantly higher (*P* < 0.00001) in camels than in other animal species (Table 2). The frequency of infection was significantly higher (*P* < 0.05) in the > 10-year-old age group than in the corresponding young group (< 6 years) (Table 3). Two infected camels that presented symptoms of progressive anemia, cachexia, dullness, and marked depression were clinically followed. One camel recovered after treatment, while the other died due to the improper administration of treatment by the owner.

## Discussion

Surra is a serious veterinary illness associated with considerable morbidity and mortality among camels in Africa, South America and Asia, including the Middle East. In Palestine, approximately 730,000 sheep, 215,000 goats, 1500 camels and 3600 equines are being raised mainly in the districts of Al-Khalil and Ariha [45]; these numbers are considered relatively low compared to those in Middle East countries, such as Saudi Arabia, Jordan and Iraq [46]. In Palestine, the overall trypanosomiasis prevalence (18%) detected was similar to the infection rate of neighboring countries in the region. Thus, Alanazi [28] has reported much lower different infection rates in donkeys (3.3%) and horses (2.8%) in Saudi Arabia. In Egypt, the prevalence was 31.4% in camels, and no infection was detected in horses and donkeys [27, 47]. In the West Bank, because the borders between Jordan and Palestine are closed, the main source of camels and other livestock is the Bedouin community in the areas in Palestine and Israel, including Al-Khalil, Ariha, Nagab Desert, Rahat and Bir-al-Saba; additionally, inbreeding occurs in Palestine. The Gaza Strip is in a similar situation, with camels imported from Egypt.

Although observing trypomastigotes, the diagnostic stage, in the peripheral blood is quite easy and straightforward, the sensitivity is low, particularly in the early stages of the disease when parasitemia is very low. Furthermore, observing parasitemia in chronic cases becomes difficult due to low and fluctuating levels. In our study, parasitemia was observed in 2.7% of the tested animals. The infection level depends on the infective dose injected by the vector and the time elapsed between ingesting the blood of an infected animal and biting an uninfected animal. *Trypanosoma evansi* does not develop in the vector like other *Trypanosoma* species but rather survives in the oral cavity of the vector [17]. Therefore, the use of molecular-based methods rather than wet mounts for *T. evansi* screening is recommended.

In the present study, we have shown that sex and age group are not risk factors for the disease, which is in congruence with other studies [28]. Surra is a vector-borne disease that provides no immunity following infection, which makes both sexes and all ages equally vulnerable to infection. The infection rate was significantly higher in camels (30%) than in the other animals, which is in agreement with the trend among livestock, as the camel is the primary host for *T. evansi* in the study area and countries of the region. However, the present study showed that sheep were also significantly infected.

Regarding future control interventions, our study showed that the main foci were in Jenin, Tubas and Ariha (Jericho); therefore, these areas need to be targeted first. The *18S* rRNA gene has been widely used as a marker for the detection of trypanosomes because it is a highly expressed multicopy gene and has formed the basis of nearly all trypanosome evolutionary analyses [48]. Nonetheless, other protein-coding genes, such as DHFR-TS (dihydrofolate reductase-thymidylate synthase); RB19 (RNA-binding protein-19), METIII (metacyclin-III), and LYT1 (lytic pathway protein), have been used in phylogenetic studies [49]. In this study, a phylogenetic and molecular analysis of the *18S* rRNA sequences has shown that one genotype of *T. evansi* was present in camels from Palestine. However, for more accurate phylogenetic analyses, it is important to sequence additional non-coding DNA regions from *T. evansi* that are genetically diverse such as the internal transcribed spacer (ITS) region [50]. A study conducted by Pourjafar et al. [50] showed that a phylogenetic analysis based on ITS2 nucleotide sequences revealed heterogeneity among the tested *T. evansi* parasites.

The clustering of all *T. evansi* samples from Palestine with those from Asia, Africa, and South America indicates the monomorphic nature of *T. evansi.* Furthermore, the grouping of all these isolates with species of *T. brucei* from Africa supports the evolution of *T. evansi* from *T. brucei* by partial or complete loss of kinetoplast DNA, supporting the hypothesis that *Trypanosoma* species originated in Africa and were spread by camels, horses and mules to Asia, Europe and South America [16, 51–53]. In the present study, we have shown that the origin of parasite isolation, whether from animals or humans, has no effect on the genetic clustering of the parasites.

## Conclusions

To the best of our knowledge this study, is the first to reveal high infection rates of surra in Palestine since 1923 [54]. The *T. evansi* population was found to be monophyletic and closely related to populations of the same species from Africa and Asia and *T. brucei* from Africa. Further studies focusing on vectors and other risk factors, such as seasonality and location, are needed to plan and develop future control programs.

## Data Availability

All data are included in the article and sequences can be accessed in GenBank. Raw data are available from the corresponding author upon request.

## Abbreviations

ELISA: enzyme-linked immunosorbent assay
VSG: variable surface glycoprotein
PCR: polymerase chain reaction
EDTA: ethylenediaminetetraacetic acid
UPGMA: unweighted pair group method with arithmetic mean
